# Outpatient drug therapy for children with heart disease in Switzerland: a survey

**DOI:** 10.1007/s00431-026-07024-z

**Published:** 2026-05-07

**Authors:** Joel Bärlocher, Angela Caduff Good, Stefano Di Bernardo, Sebastiano A. G. Lava, Dominik Stambach, Cécile Tissot, Walter Knirsch, Julie Wacker, Anna Cavigelli-Brunner, Alessandra Bosch, Christian Balmer

**Affiliations:** 1 Division of Paediatric Cardiology, Paediatric Heart Center, Department of Surgery, University Children’s Hospital Zurich, Zurich, Switzerland; 2 Children’s Research Center, University Children’s Hospital Zurich, Zurich, Switzerland; 3 Hospital Pharmacy, University Children’s Hospital Zurich, Zurich, Switzerland; 4 Paediatric Cardiology, Department of Paediatrics, Lausanne University Hospital, Lausanne, Switzerland; 5 Centre Universitaire Romand de Cardiologie Et Chirurgie Cardiaque Pédiatrique, University Hospitals of Geneva, Geneva, Switzerland; 6 Swiss Pediatric Heart Research Association, Zurich, Switzerland; 7 Paediatric Cardiology, Children’s Hospital of Eastern Switzerland, St. Gallen, St. Gallen, Switzerland; 8 Department of Paediatrics, Chêne-Bougeries, Clinique Des Grangettes, Geneva, Switzerland; 9 Paediatric Cardiology Unit, Faculty of Medicine, University Hospitals of Geneva, University of Geneva, Geneva, Switzerland; 10 Department of Haematology, University Children’s Hospital Zurich, Zurich, Switzerland

**Keywords:** Heart failure, Arrhythmia, Thromboembolism, Drug therapy, Pharmacotherapy

## Abstract

Cardiovascular drug therapy in adults is steadily progressing. However, the extent to which children with heart disease in Switzerland benefit from this progress remains unknown. This survey aimed to investigate the current outpatient prescribing practices among paediatric cardiologists in Switzerland. We conducted a cross-sectional survey among Swiss paediatric cardiologists. The physicians were asked to state how often they administer drugs on a pre-defined list for the following indications: heart failure, arrhythmia and thromboembolism. Forty-three (56%) out of 77 eligible physicians completed the survey. For paediatric heart failure, the three most frequently prescribed drugs were hydrochlorothiazide (‘often prescribed’ by *n* = 25, 58%), lisinopril (*n* = 24, 56%), and spironolactone (*n* = 32, 74%). The most frequently prescribed drugs for arrhythmia and thromboembolism were propranolol (*n* = 26, 60%) and acetylsalicylic acid (*n* = 31, 72%), respectively. Newer drugs such as sacubitril/valsartan (‘never prescribed’ by *n* = 20, 47%), sodium-glucose cotransporter type 2 inhibitors (*n* = 40, 93%), and direct oral anticoagulants (*n* = 20, 47%) were rarely used.

*Conclusion:* The drugs used to treat heart failure, arrhythmia, and thromboembolism in Switzerland largely reflect current international recommendations. Nevertheless, novel drugs are rarely used. In addition to the lack of large randomized controlled drug trials in children, important obstacles are lack of authorization, lack of reimbursement, and lack of suitable formulations. These issues should be addressed by both the industry and healthcare providers to continuously improve drug therapy for children with heart disease.

**Table:** 

**What is Known:** • *Evidence for drug use in Paediatric cardiology mostly relies on extrapolation from adult studies and pathophysiological considerations*. • *Drug choices are also influenced by drug approval, insurance reimbursement, and availability of age-appropriate formulations*.	
**What is New:** • *Swiss paediatric cardiologists predominantly rely on established drugs to treat cardiovascular diseases in children, with the use of newer drugs remaining limited*. • *On top of expert consensus, adult evidence, and drug availability, drug prescription appears to be strongly influenced by the availability of child-friendly formulations*.

**Supplementary Information:**

The online version contains supplementary material available at https://doi.org/10.1007/s00431-026-07024-z.

## Introduction

Drugs play an important role in the treatment of children with cardiovascular diseases. They are used to control heart failure, arrhythmias, and thromboembolism. Pharmacological research has provided the basis for many of the recent advances in medical care, with particular benefits for patients with cardiovascular diseases [1]. However, many drugs lack formal studies in children, and as many as 88% of paediatric outpatients with heart disease receive at least one off-label drug [2]. Treating physicians face the choice of either neglecting drugs not yet approved for children and provide ‘nihilistic’ healthcare [3] or treating their patients with off-label drugs, in the hope that these will have similar benefits and side-effect profiles as in their approved population (i.e., adults). This is suboptimal, since drug effects and pharmacokinetics can differ in children as compared to adults [4]. In addition, paediatric clinical setting may be peculiar: 1) the most common causes of heart failure in children are congenital heart defects and cardiomyopathies, whereas in adults ischemia and arterial hypertension prevail [5]. 2) The causes and spectrum of cardiac arrhythmias are age specific. For example, atrial fibrillation is rare in children but very common in adults [6]. 3) The same is true for antithrombotic and anticoagulant therapy: the focus in children is on patients with an underlying congenital heart disease, such as Fontan physiology, or on postoperative and postinterventional therapy [7], whereas in adults, the focus is mainly on patients with atrial fibrillation or coronary heart disease [8, 9].

Prescription practices of drugs in children with cardiac diseases in Switzerland are currently unknown. The aim of this survey was to investigate current outpatient prescription practices of Swiss paediatric cardiologists.

## Materials and methods

### Study design

An online cross-sectional survey was conducted among Swiss paediatric cardiologists between June and October 2023. The survey was developed and reported according to the checklist for reporting of survey studies (CROSS) [10].

### Data collection methods

The questionnaire consisted of five sections. In the first three sections, a list of drugs was provided, and survey participants were asked how often they estimate prescribing each listed drug for heart failure, arrhythmia, and thromboembolism, respectively. The estimated frequency of administration for each drug was indicated on a 4-point Likert scale with the answer options ‘never’, ‘seldom’, ‘sometimes’, and ‘often’ (estimated number of patients treated with the listed drug). A free text option was also offered to name drugs that were not present in the provided list. In the fourth section of the questionnaire, survey participants were asked to rate, on a 5-point Likert scale with answer options from ‘very poor’ to ‘excellent’, their impression on the current pharmacotherapeutic situation for children with heart disease in Switzerland. A free text field was additionally provided. In the final section, demographic data was gathered (e.g. working setting, years of experience in paediatric cardiology). Throughout the survey, participants were asked to specifically and only refer to outpatient oral therapy. The survey was pilot-tested with two paediatric cardiologists to ensure the clarity of the questionnaire and to assess the time required to complete it.

### Sample characteristics

Study respondents were paediatric cardiologists practising in Switzerland. A list of eligible respondents was provided by the Swiss Society of Paediatric Cardiology, which includes the vast majority of Swiss paediatric cardiologists. A total of 77 physicians were invited.

### Survey administration

Potential survey participants were contacted by email on 20 June 2023. The LimeSurvey online tool available from the University of Zurich was used. Two weeks later, a reminder was sent.

### Ethical considerations

This survey was exempt from ethics approval. The answering of the questionnaire was deemed as implicit consent to participate in the anonymous survey.

### Statistical analysis

To analyse associations between working setting, years of experience, and prescribing patterns, the Mann–Whitney test was employed. To facilitate comparison between more and less experienced paediatric cardiologists, respondents were divided into two similarly sized groups by their years of experience in paediatric cardiology. Statistical significance was set at *p* < 0.05. We used R version 4.3.1 [11] and the following R packages: flextable version 0.9.6 [12], kableExtra version 1.4.0 [13], officer version 0.6.6 [14], pacman version 0.5.1 [15], rio version 1.0.1 [16], and tidyverse version 2.0.0 [17].

## Results

### Respondent characteristics

Forty-three (56%) out of 77 invited paediatric cardiologists fully answered the questionnaire. Two further respondents only completed the survey partially, and their responses were thus excluded from further analysis. Demographic characteristics of the respondents are summarized in Table 1. Their median years of experience in paediatric cardiology was 15 years (range: 4 to > 30 years).

**Table 1:** Demographic characteristics of survey respondents

Characteristic	n (%)
**Years of experience in paediatric cardiology**	
< 10	7 (16.3)
10—20	28 (65.1)
> 20	8 (18.6)
**Facility**	
Hospital with paediatric heart surgery	22 (51.2)
Hospital without paediatric heart surgery	12 (27.9)
Private practice	9 (20.9)

### Heart failure

The most frequently prescribed drugs for heart failure, with a median frequency of ‘often’, were lisinopril, hydrochlorothiazide, and spironolactone (Table 2, Supplementary Table S1). Spironolactone emerged as the most frequently prescribed heart failure drug, with 74% of respondents reporting that they often use it. In the free text field, ivabradine and coenzyme Q10 were each stated once to be seldom used.

**Table 2:** Median frequency of prescription of heart failure drugs

Often (%)	Sometimes (%)	Seldom (%)	Never (%)^a^		
Lisinopril (56)	Captopril (40)	Enalapril (30)	Cilazapril (98)	Telmisartan (100)	Indapamide (98)
Hydrochlorothiazide (58)	Bisoprolol (35)	Losartan (33)	Perindopril (81)	Valsartan (67)	Metolazone (88)
Spironolactone (74)	Propranolol (21)	Atenolol (40)	Quinapril (100)	Celiprolol (98)	Tolvaptan (88)
	Furosemide (28)	Carvedilol (35)	Ramipril (81)	Labetalol (93)	Canagliflozin (100)
		Metoprolol (35)	Azilsartan (98)	Nadolol (72)	Dapagliflozin (93)
		Digoxin (42)	Candesartan (70)	Nebivolol (81)	Empagliflozin (93)
		Torasemide (16)	Eprosartan (100)	Sotalol (65)	Ertugliflozin (100)
		Sacubitril/Valsartan (40)	Irbesartan (91)	Eplerenone (84)	
			Olmesartan (95)	Finerenone (98)	

^a^ Median frequency of ‘never’ indicates that more than half of all respondents never prescribe the drug specified. It does not necessarily mean that none of the respondents ever prescribe that drug. For more detailed results, see appendix

### Arrhythmia

Propranolol was the most frequently prescribed antiarrhythmic drug, with 60% of respondents prescribing it often (Table 3, Supplementary Table S2). Among all drugs in the questionnaire, propafenone was the only one for which physicians with more than 15 years of experience in paediatric cardiology administered it more frequently than their less experienced colleagues (*p* = 0.0007). In the free text field, magnesium was mentioned by one respondent, who stated to administer it sometimes.

**Table 3:** Median frequency of prescription of antiarrhythmics

Often (%)	Sometimes (%)	Seldom (%)	Never (%)^a^
Propranolol (70)	Flecainide (49)	Propafenone (37)*	Carvedilol (51)
	Bisoprolol (44)	Atenolol (42)	Celiprolol (100)
	Metoprolol (58)	Sotalol (14)	Labetalol (95)
	Amiodarone (30)	Digoxin (40)	Nadolol (63)
			Nebivolol (88)
			Dronedarone (98)
			Diltiazem (79)
			Verapamil (67)
			Ivabradine (65)

^a^ Median frequency of ‘never’ indicates that more than half of all respondents never prescribe the drug specified. It does not necessarily mean that none of the respondents ever prescribe that drug. For more detailed results, see appendix

^*^ Prescribed more frequently by physicians with > 15 years of experience in paediatric cardiology (*p* = 0.0007)

### Thromboembolism

Among anticoagulants and antiplatelet drugs, acetylsalicylic acid (ASA) was the most frequently used, being often prescribed by 72% of respondents (Table 4, Supplementary Table S3). No other drugs were mentioned in the free text field.

**Table 4:** Median frequency of prescription of anticoagulants and antiplatelet drugs

Often (%)	Sometimes (%)	Seldom (%)	Never (%)^a^
Acetylsalicylic Acid (72)	Phenprocoumon (35)	Clopidogrel (21)	Prasugrel (95)
		Apixaban (26)	Ticagrelor (95)
			Dabigatran (95)
			Edoxaban (98)
			Rivaroxaban (63)
			Acenocoumarol (70)

^a^ Median frequency of ‘never’ indicates that more than half of all respondents never prescribe the drug specified. It does not necessarily mean that none of the respondents ever prescribe that drug. For more detailed results, see appendix

### Perception of the current pharmacotherapeutic situation

**Fig. 1:**
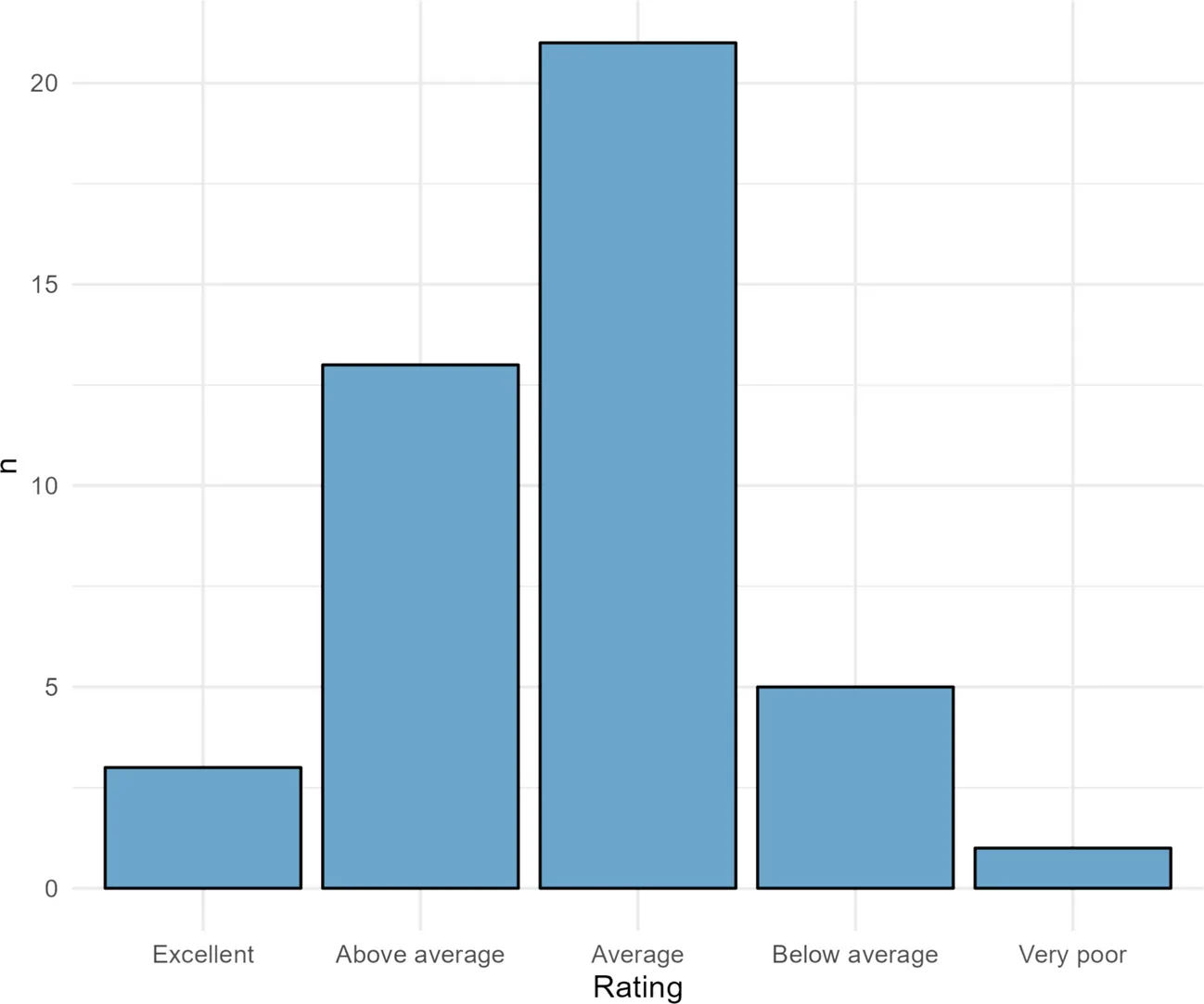
Rating of the current pharmacotherapeutic situation in children with heart disease in Switzerland (n = 43)

The current pharmacotherapeutic situation for children with heart disease in Switzerland was perceived as average or better by most respondents (Fig. 1). In the free text field, two respondents expressed the wish for the approval of nadolol by the national competent authority (Swissmedic) for the treatment of patients with long QT syndrome, reducing administrative struggles. Other paediatricians reported that they frequently prescribe off-label drugs, and another two expressed the wish for a consensus and updated guidance on the treatment of heart failure. Some respondents reported that formulations for children are scarce and difficult to obtain, and that the evidence base for paediatric cardiovascular therapy is globally limited and requires further studies. One participant stressed the benefit of pharmacies being able to manufacture individually dosed, child-sized capsules or liquid formulations. One respondent noted that ‘many drugs are available and would be of benefit in children. Paediatricians and paediatric cardiologists seem to be reluctant in adopting “new” drugs’.

## Discussion

This survey provides an overview of drug prescriptions as stated by participating Swiss paediatric cardiologists. This shows a reliance on established treatments such as angiotensin-converting-enzyme (ACE) inhibitors, beta-adrenergic receptor (AR) antagonists and ASA, while novel drugs, such as sodium-glucose cotransporter 2 (SGLT2) inhibitors, sacubitril/valsartan, and direct oral anticoagulants (DOACs), are rarely used.

At 56%, the response rate to this survey was good. Moreover, the respondents included a high proportion of physicians with many years of professional experience and a balanced proportion of physicians at hospitals with or without cardiac surgery, and from private practices. Consequently, we consider the survey to be representative of Swiss paediatric cardiologists.

### Heart failure

ACE inhibitors, diuretics, and beta blockers are the preferred drugs for heart failure therapy in children. In contrast, digoxin, combined alpha and beta-AR antagonists, angiotensin II receptor antagonists, angiotensin receptor/neprilysin inhibitors, and SGLT2 inhibitors were reported to be rarely prescribed.

The primary use of ACE inhibitors, diuretics, and beta-blockers reflects the few recommendations available for paediatric heart failure [18–20].

Of note, these recommendations are based on expert consensus and extrapolation from data available in adults. Consequently, the impact of pharmacological therapies on the unique pathophysiology and anatomy of various congenital heart lesions and the pharmacokinetic and pharmacodynamic peculiarities of children, including their ontogeny along growth and development, should be carefully and individually considered [4, 21].

Hydrochlorothiazide was reported to be the most frequently prescribed diuretic, with 58% of respondents stating to often administer it. Thiazide diuretics are generally recommended for use in paediatric heart failure and can synergistically enhance the diuretic effect of loop diuretics in chronic use [21–23]. Epidemiological studies in the Danish population have suggested that the known photosensitizing effect of hydrochlorothiazide might increase the risk of both non-melanoma skin cancer and melanoma in the elderly [23, 24]. These studies have several caveats [25], and their impact on Paediatrics is unclear. However, it seems prudent to use some caution whilst prescribing long-term hydrochlorothiazide. Most importantly, patients and parents must be informed about the key necessity of consequent photoprotection.

A high number of respondents (40%) indicated that they often use propranolol, a nonselective beta-AR antagonist, for the treatment of heart failure, with no significant difference in prescription frequency from bisoprolol, a beta-1-selective AR blocker (*p* = 0.13). One might have expected that beta-1-selective AR blockers would mostly be used for the treatment of heart failure, since they have a cardioprotective effect and do not lead to bronchial obstruction [3, 26–28]. Given that propranolol is frequently used in infants and children for various indications including arrhythmias and outflow tract obstructions, it is possible that some respondents might have reported their prescription frequency across all indications and not exclusively for heart failure. Also, in paediatric clinical practice, propranolol is sometimes preferred because of its shorter half-life compared to bisoprolol. The availability of a liquid formulation for propranolol might also contribute to shape the prescription preferences of practicing paediatric cardiologists.

In 2014, sacubitril/valsartan was introduced as a new heart failure drug in adults [29]. Despite the U.S. Food and Drug Administration’s approval of sacubitril/valsartan for certain paediatric heart failure patients in 2019 based on the PANORAMA-HF study [30], according to this survey its use remains limited in Switzerland. This may be due to the lack of authorization for children in Switzerland and the challenges associated with its administration in young paediatric patients due to the galenic form. Of note, a child-adequate formulation (not available at the time of the survey) has meanwhile been developed.

SGLT2 inhibitors are currently very rarely used to treat paediatric heart failure in Switzerland. Research on adult patients has shown that the SGLT2 inhibitors dapagliflozin and empagliflozin lower the risk of cardiovascular death and hospitalizations in a wide range of heart failure patients, both with reduced and preserved ejection fraction [31, 32]. Although little information is available on paediatric use of this class of drugs, use of dapagliflozin and empagliflozin in children has so far shown good tolerability and encouraging results [33, 34].

### Arrhythmia

The most common arrhythmia in paediatric patients is supraventricular tachycardia (SVT), especially accessory pathway-mediated tachycardia and atrioventricular nodal re-entrant tachycardia [35]. A Europe-wide survey identified atenolol (a beta-blocker) and flecainide (a sodium channel blocker) as the preferred drugs for recurrent SVTs in children. Although that survey did not specifically address paediatric cardiologists [36], we detected a similar pattern in our survey: beta-blockers were the most frequently prescribed drugs, with propranolol, bisoprolol, and metoprolol as preferred active substances.

The reason why 60% of respondents use propranolol frequently is probably its safety profile and the availability of a liquid formulation, making it convenient for individualized dose adjustment even in infants. Children with electric cardiomyopathies such as long QT syndrome benefit from prophylactic nonselective beta-blockers such as propranolol [37]. For long QT syndrome, nadolol has the advantage of its long half-life and therefore better compliance [38]. Unfortunately, it is not authorized in Switzerland and it is sometimes not reimbursed by health insurances. It is therefore unsurprising that, according to our survey, nadolol is rarely administered and that some respondents specifically asked for its authorization.

Flecainide is a class IC antiarrhythmic and is primarily recommended for the management of SVT in children [39, 40]. The efficacy of propafenone (from the same class) is similar in the setting of SVT in children [40]. However, it was reported to be used less frequently than flecainide, and it is noticeable that propafenone was reportedly used more frequently by physicians with professional experience in paediatric cardiology of over 15 years. Indeed, propafenone 10 mg tablets used to be available, allowing practical and precise dosing for children, up to 2015, when this formulation was withdrawn from the Swiss market. At the same time, a flecainide suspension was introduced. This example demonstrates how drug formulation availability is a key factor whilst selecting a paediatric drug.

### Thromboembolism

Platelet inhibition is indicated in children with several heart diseases, including Kawasaki syndrome, surgical aorto-pulmonary shunts, Fontan circulation, and presence of intracardiac devices [41]. In our survey, ASA was the most frequently used platelet inhibitor.

Recently, paediatric clinical trials with DOACs have been conducted in children with heart disease. These trials compared rivaroxaban, apixaban, and edoxaban to the standard of care, which includes vitamin K antagonists (VKAs) and low-molecular-weight heparins (LMWHs), with or without ASA, for thromboprophylaxis in a variety of congenital and acquired heart diseases. Overall, these trials showed safety and efficacy equivalent to the standard of care [42–45]. Additionally, real-world data is emerging that supports the use of apixaban and rivaroxaban for thromboprophylaxis in this population [46, 47]. DOACs offer a number of benefits over VKAs: predictable pharmacokinetics, independence from antithrombin, minimal food and drug interactions, an extended therapeutic range, and potentially no necessity for routine monitoring with painful blood tests [48]. These benefits make DOACs an attractive alternative to VKAs in paediatrics.

At the time of the survey, none of the DOACs were licensed for thromboprophylaxis among children in Switzerland. This could explain why survey respondents stated rarely to use them. Interestingly, also VKAs are off-label drugs in Paediatrics, but they are more frequently used than DOACs. This might be because of the long-lasting experience with these older drugs.

### Limitations

This study being conducted among highly specialized physicians in a small country, the number of eligible respondents is small. Nonetheless, the response rate of 56% is relatively high compared to other web-based surveys among physicians [49, 50].

The number of patients that were treated with a specific drug was not measured but estimated and reported by the respondents. Thus, there is an intrinsic risk of recall bias.

Another limitation is that the indications for medication use were broad. Because various types of heart failure and arrhythmias require different treatments, this prevents us from drawing conclusions about specific conditions. Moreover and importantly, the questionnaire did not allow any answers regarding combinations of drugs. Considering that heart failure and some difficult arrhythmias are typically treated with multiple drugs, this does not allow to draw conclusions about preferred combination therapies.

Unfortunately, the reasons behind specific drug choices were not elicited by this survey, so that results interpretation is based on the ‘free text’ comments and our own hypothesis and therefore remains somewhat speculative. Potential reasons may include, among others, availability, costs/health-insurance reimbursement, expected adherence, palatability, known side effects, ease of monitoring, and may be more pronounced in patients with multiple drugs.

In retrospect, we acknowledge that the use of real-life scenarios (instead of or in addition to general questions) might have allowed us to gain better insights in the attitudes of everyday clinical life.

Finally, the statistical comparisons (e.g. more versus less years of experience in paediatric cardiology) are based on small-sampled subgroups and therefore needs to be interpreted with caution.

## Conclusions

Drug therapy for cardiovascular diseases in children in Switzerland is mainly accomplished with traditional drugs. This survey suggests that, on top of available evidence (mostly extrapolated from adult studies), prescribing practices are also influenced by drugs’ licensing and availability, financial reimbursement, and the availability of age-appropriate formulations.

As expected, the adoption of novel drugs takes time to generate evidence, get approval, and change prescribing practices. There is a potential for further improvement in the drug therapy of cardiovascular diseases in children. Pharmaceutical industry, researchers, national authorities, clinicians and the public have a shared responsibility to continuously evaluate new drugs’ benefits and risks in paediatric patients.

## Supplementary Information

Below is the link to the electronic supplementary material.

ESM 1 (DOCX 39.1 KB)

ESM 2 (PDF 386 KB)

## Data Availability

The data that support the findings of this study are available upon reasonable request.

## Code availability

The code used in this study is available upon reasonable request.
