# Supplementary material for: Outpatient drug therapy for children with heart disease in Switzerland: a survey
Source: Eur J Pediatr. 2026 May 7;185(6):361. doi: 10.1007/s00431-026-07024-z (PMC13652761; doi:10.1007/s00431-026-07024-z)
Supplement: Supplementary file 1 [file 431_2026_7024_MOESM2_ESM.pdf]

0%

This survey is currently not active. You will not be able to save your responses. ×

Survey on the administration of oral medication in children with heart disease in Switzerland

The aim of this survey is to find out which drugs are administered for three different indications (1. heart failure, 2. arrhythmia, 3. coagulation) in children with cardiovascular diseases in Switzerland.

You will be asked to state how often you administer a certain drug for the indications mentioned above. For example, if you treat five patients per year with heart failure and you administer drug X in all of these five cases, drug X can be considered to be administered often. However, if you treat 250 patients per year with heart failure, treating five of these patients with drug X can no longer be considered as often. Hence, the frequency of administering a certain drug depends on your patient collective.

Throughout the survey, please refer only to **outpatient therapy** using **peroral administration** (tablets, capsules, granules, powders, solutions, emulsions, suspensions, etc.).

The survey takes about 5 minutes to complete.

Next

0%

This survey is currently not active. You will not be able to save your responses. ×

1. Heart failure

- Please state which of the following active substances you administer for the treatment of heart failure and how often.
- Please refer only to an outpatient setting using peroral administration.

* ACE Inhibitors

	Never	Seldom	Sometimes	Often
Captopril	○	○	○	○
Cilazapril (e.g. Inhibace)	○	○	○	○
Enalapril (e.g. Epril)	○	○	○	○
Lisinopril (e.g. Zestril)	○	○	○	○
Perindopril (e.g. Coversum)	○	○	○	○
Quinapril (e.g. Accupro)	○	○	○	○
Ramipril (e.g. Triatec)	○	○	○	○

✱ Angiotensin II Receptor Blockers

	Never	Seldom	Sometimes	Often
Azilsartan (e.g. Edarbi)	◯	◯	◯	◯
Candesartan (e.g. Atacand)	◯	◯	◯	◯
Eprosartan (e.g. Teveten)	◯	◯	◯	◯
Irbesartan (e.g. Aprovel)	◯	◯	◯	◯
Losartan (e.g. Cosaar)	◯	◯	◯	◯
Olmesartan (e.g. Olmetec)	◯	◯	◯	◯
Telmisartan (e.g. Micardis)	◯	◯	◯	◯
Valsartan (e.g. Diovan)	◯	◯	◯	◯

✱ Beta-Adrenoceptor Antagonists

	Never	Seldom	Sometimes	Often
Atenolol (e.g. Tenormin)	◯	◯	◯	◯
Bisoprolol (e.g. Concor)	◯	◯	◯	◯
Carvedilol (e.g. Dilatrend)	◯	◯	◯	◯
Celiprolol (e.g. Selectol)	◯	◯	◯	◯
Labetalol (e.g. Trandate)	◯	◯	◯	◯
Metoprolol (e.g. Beloc ZOK)	◯	◯	◯	◯
Nadolol	◯	◯	◯	◯
Nebivolol (e.g. Nebilet)	◯	◯	◯	◯
Propranolol (e.g. Inderal)	◯	◯	◯	◯
Sotalol	◯	◯	◯	◯

✱ Cardiac Glycosides

	Never	Seldom	Sometimes	Often
Digoxin	◯	◯	◯	◯

✱ Diuretics

	Never	Seldom	Sometimes	Often
Eplerenon (e.g. Inspra)	◯	◯	◯	◯
Finerenon (e.g. Kerendia)	◯	◯	◯	◯
Furosemid (e.g. Lasix)	◯	◯	◯	◯
Hydrochlorothiazid (e.g. Esidrex)	◯	◯	◯	◯
Indapamid (e.g. Fludex)	◯	◯	◯	◯
Metolazon	◯	◯	◯	◯
Spironolacton (e.g. Aldactone)	◯	◯	◯	◯
Tolvaptan (e.g. Jinarc)	◯	◯	◯	◯
Torasemid (e.g. Torem)	◯	◯	◯	◯

✱ SGLT2 Inhibitors

	Never	Seldom	Sometimes	Often
Canagliflozin (e.g. Invokana)	◯	◯	◯	◯
Dapagliflozin (e.g. Forxiga)	◯	◯	◯	◯
Empagliflozin (e.g. Jardiance)	◯	◯	◯	◯

	Never	Seldom	Sometimes	Often
Ertugliflozin (e.g. Steglatro)	○	○	○	○

✱ Combination Drugs

	Never	Seldom	Sometimes	Often
Sacubitril/Valsartan (Entresto)	○	○	○	○

Others

Never

Seldom

Sometimes

Often

Previous

Next

16%

This survey is currently not active. You will not be able to save your responses. ×

2. Arrhythmia

- Please state which of the following active substances you administer for the treatment of arrhythmia and how often.
- Please refer only to an outpatient setting using peroral administration.

✱ Antiarrhythmic Agents Class I

	Never	Seldom	Sometimes	Often
Flecainid (e.g. Tambocor)	◯	◯	◯	◯
Propafenon (e.g. Rytmonorm)	◯	◯	◯	◯

✱ Antiarrhythmic Agents Class II

	Never	Seldom	Sometimes	Often
Atenolol (e.g. Tenormin)	◯	◯	◯	◯
Bisoprolol (e.g. Concor)	◯	◯	◯	◯

	Never	Seldom	Sometimes	Often
Carvedilol (e.g. Dilatrend)	◯	◯	◯	◯
Celiprolol (e.g. Selectol)	◯	◯	◯	◯
Labetalol (e.g. Trandate)	◯	◯	◯	◯
Metoprolol (e.g. Beloc ZOK)	◯	◯	◯	◯
Nadolol	◯	◯	◯	◯
Nebivolol (e.g. Nebilet)	◯	◯	◯	◯
Propranolol (e.g. Inderal)	◯	◯	◯	◯
Sotalol	◯	◯	◯	◯

✱ Antiarrhythmic Agents Class III

	Never	Seldom	Sometimes	Often
Amiodaron (e.g. Cordarone)	◯	◯	◯	◯
Dronedaron (e.g. Multaq)	◯	◯	◯	◯

✱ Antiarrhythmic Agents Class IV

	Never	Seldom	Sometimes	Often
Diltiazem (e.g. Dilzem)	◯	◯	◯	◯
Verapamil (e.g. Isoptin)	◯	◯	◯	◯

✱ Other Antiarrhythmic Agents

	Never	Seldom	Sometimes	Often
Digoxin	◯	◯	◯	◯
Ivabradin (e.g. Procoralan)	◯	◯	◯	◯

Others

Never

Seldom

Sometimes

Often

Previous

Next

33%

This survey is currently not active. You will not be able to save your responses. ×

3. Coagulation

- Please state which of the following active substances you administer for antithrombosis and how often.
- Please refer only to an outpatient setting using peroral administration.

✱ Antiplatelet Drugs

	Never	Seldom	Sometimes	Often
Acetylsalicylic Acid (e.g. Aspirin)	◯	◯	◯	◯
Clopidogrel (e.g. Plavix)	◯	◯	◯	◯
Prasugrel (e.g. Efient)	◯	◯	◯	◯
Selexipag (e.g. Uptravi)	◯	◯	◯	◯
Ticagrelor (e.g. Brilique)	◯	◯	◯	◯

✱ Direct Oral Anticoagulants

	Never	Seldom	Sometimes	Often
Apixaban (e.g. Eliquis)	◯	◯	◯	◯
Dabigatran etexilat (e.g. Pradaxa)	◯	◯	◯	◯
Edoxaban (e.g. Lixiana)	◯	◯	◯	◯
Rivaroxaban (e.g. Xarelto)	◯	◯	◯	◯

✱ Vitamin K Antagonists

	Never	Seldom	Sometimes	Often
Acenocoumarol (e.g. Sintrom)	◯	◯	◯	◯
Phenprocoumon (e.g. Marcoumar)	◯	◯	◯	◯

Others

Never

Seldom

Sometimes

Often

50%

This survey is currently not active. You will not be able to save your responses. ×

Current pharmacotherapeutic situation in children with heart disease in Switzerland

- Please share with us your experience and your personal opinion.
- Please refer only to an outpatient setting using peroral administration.

* How do you rate the current pharmacotherapeutic situation in children with heart disease in Switzerland?

An additional comment is optional.

Choose one of the following answers

○ Excellent

○ Above average

○ Average

○ Below average

○ Very Poor

Please enter your comment here:

Previous

Next

66%

This survey is currently not active. You will not be able to save your responses. ×

General Informations

✱ Please state in which type of medical facility you are currently working.

❗ Check all that apply

☐ Hospital with children heart surgery

☐ Hospital without children heart surgery

☐ Private practice

✱ Please state how many years of professional experience you have in paediatric cardiology.

If you wish to be informed about the outcomes of the study, please state your name and email address. (optional)

Name

Email address

Previous

Next

83%

This survey is currently not active. You will not be able to save your responses.

×

Further comments (optional)

Previous

Submit
