# Supplementary material for: Outpatient drug therapy for children with heart disease in Switzerland: a survey
Source: Eur J Pediatr. 2026 May 7;185(6):361. doi: 10.1007/s00431-026-07024-z (PMC13652761; doi:10.1007/s00431-026-07024-z)
Supplement: Supplementary file 2 [file 431_2026_7024_MOESM1_ESM.docx]

## Appendix

**Supplementary Table 1**

Frequency of prescription of heart failure drugs (n = 43)

| **Drug** | **Often n % (95% CI)** | **Sometimes n % (95% CI)** | **Seldom n % (95% CI)** | **Never n % (95% CI)** | **Median** |
| --- | --- | --- | --- | --- | --- |
| Captopril | 17 40 (24.9-54.1) | 17 40 (24.9-54.1) | 6 14 (3.6-24.3) | 3 7 (0-14.6) | Sometimes |
| Cilazapril | 0 0 (0-0) | 0 0 (0-0) | 1 2 (0-6.8) | 42 98 (93.2-100) | Never |
| Enalapril | 6 14 (3.6-24.3) | 15 35 (20.6-49.1) | 13 30 (16.5-44) | 9 21 (8.8-33.1) | Seldom |
| Lisinopril | 24 56 (41-70.7) | 10 23 (10.6-35.9) | 2 5 (0-10.9) | 7 16 (5.2-27.3) | Often |
| Perindopril | 3 7 (0-14.6) | 2 5 (0-10.9) | 3 7 (0-14.6) | 35 81 (69.8-93) | Never |
| Quinapril | 0 0 (0-0) | 0 0 (0-0) | 0 0 (0-0) | 43 100 (100-100) | Never |
| Ramipril | 0 0 (0-0) | 2 5 (0-10.9) | 6 14 (3.6-24.3) | 35 81 (69.8-93) | Never |
| Azilsartan | 0 0 (0-0) | 0 0 (0-0) | 1 2 (0-6.8) | 42 98 (93.2-100) | Never |
| Candesartan | 1 2 (0-6.8) | 7 16 (5.2-27.3) | 5 12 (2-21.2) | 30 70 (56-83.5) | Never |
| Eprosartan | 0 0 (0-0) | 0 0 (0-0) | 0 0 (0-0) | 43 100 (100-100) | Never |
| Irbesartan | 0 0 (0-0) | 1 2 (0-6.8) | 3 7 (0-14.6) | 39 91 (82-99.4) | Never |
| Losartan | 5 12 (2-21.2) | 10 23 (10.6-35.9) | 14 33 (18.6-46.6) | 14 33 (18.6-46.6) | Seldom |
| Olmesartan | 0 0 (0-0) | 1 2 (0-6.8) | 1 2 (0-6.8) | 41 95 (89.1-100) | Never |
| Telmisartan | 0 0 (0-0) | 0 0 (0-0) | 0 0 (0-0) | 43 100 (100-100) | Never |
| Valsartan | 1 2 (0-6.8) | 7 16 (5.2-27.3) | 6 14 (3.6-24.3) | 29 67 (53.4-81.4) | Never |
| Atenolol | 2 5 (0-10.9) | 5 12 (2-21.2) | 17 40 (24.9-54.1) | 19 44 (29.3-59) | Seldom |
| Bisoprolol | 20 47 (31.6-61.4) | 15 35 (20.6-49.1) | 5 12 (2-21.2) | 3 7 (0-14.6) | Sometimes |
| Carvedilol | 5 12 (2-21.2) | 15 35 (20.6-49.1) | 15 35 (20.6-49.1) | 8 19 (7-30.2) | Seldom |
| Celiprolol | 0 0 (0-0) | 0 0 (0-0) | 1 2 (0-6.8) | 42 98 (93.2-100) | Never |
| Labetalol | 0 0 (0-0) | 1 2 (0-6.8) | 2 5 (0-10.9) | 40 93 (85.4-100) | Never |
| Metoprolol | 6 14 (3.6-24.3) | 13 30 (16.5-44) | 15 35 (20.6-49.1) | 9 21 (8.8-33.1) | Seldom |
| Nadolol | 2 5 (0-10.9) | 5 12 (2-21.2) | 5 12 (2-21.2) | 31 72 (58.7-85.5) | Never |
| Nebivolol | 0 0 (0-0) | 2 5 (0-10.9) | 6 14 (3.6-24.3) | 35 81 (69.8-93) | Never |
| Propranolol | 17 40 (24.9-54.1) | 9 21 (8.8-33.1) | 8 19 (7-30.2) | 9 21 (8.8-33.1) | Sometimes |
| Sotalol | 1 2 (0-6.8) | 4 9 (0.6-18) | 10 23 (10.6-35.9) | 28 65 (50.9-79.4) | Never |
| Digoxin | 1 2 (0-6.8) | 6 14 (3.6-24.3) | 18 42 (27.1-56.6) | 18 42 (27.1-56.6) | Seldom |
| Eplerenone | 0 0 (0-0) | 3 7 (0-14.6) | 4 9 (0.6-18) | 36 84 (72.7-94.8) | Never |
| Finerenone | 0 0 (0-0) | 0 0 (0-0) | 1 2 (0-6.8) | 42 98 (93.2-100) | Never |
| Furosemide | 20 47 (31.6-61.4) | 12 28 (14.5-41.3) | 7 16 (5.2-27.3) | 4 9 (0.6-18) | Sometimes |
| Hydrochlorothiazide | 25 58 (43.4-72.9) | 10 23 (10.6-35.9) | 7 16 (5.2-27.3) | 1 2 (0-6.8) | Often |
| Indapamide | 0 0 (0-0) | 0 0 (0-0) | 1 2 (0-6.8) | 42 98 (93.2-100) | Never |
| Metolazone | 0 0 (0-0) | 0 0 (0-0) | 5 12 (2-21.2) | 38 88 (78.8-98) | Never |
| Spironolactone | 32 74 (61.4-87.5) | 9 21 (8.8-33.1) | 2 5 (0-10.9) | 0 0 (0-0) | Often |
| Tolvaptan | 0 0 (0-0) | 1 2 (0-6.8) | 4 9 (0.6-18) | 38 88 (78.8-98) | Never |
| Torasemide | 5 12 (2-21.2) | 13 30 (16.5-44) | 7 16 (5.2-27.3) | 18 42 (27.1-56.6) | Seldom |
| Canagliflozin | 0 0 (0-0) | 0 0 (0-0) | 0 0 (0-0) | 43 100 (100-100) | Never |
| Dapagliflozin | 0 0 (0-0) | 0 0 (0-0) | 3 7 (0-14.6) | 40 93 (85.4-100) | Never |
| Empagliflozin | 0 0 (0-0) | 0 0 (0-0) | 3 7 (0-14.6) | 40 93 (85.4-100) | Never |
| Ertugliflozin | 0 0 (0-0) | 0 0 (0-0) | 0 0 (0-0) | 43 100 (100-100) | Never |
| Sacubitril Valsartan | 0 0 (0-0) | 6 14 (3.6-24.3) | 17 40 (24.9-54.1) | 20 47 (31.6-61.4) | Seldom |

**Supplementary Table 2**

Frequency of prescription of antiarrhythmics (n = 43)

| **Drug** | **Often n % (95% CI)** | **Sometimes n % (95% CI)** | **Seldom n % (95% CI)** | **Never n % (95% CI)** | **Median** |
| --- | --- | --- | --- | --- | --- |
| Flecainide | 10 23 (10.6-35.9) | 21 49 (33.9-63.8) | 9 21 (8.8-33.1) | 3 7 (0-14.6) | Sometimes |
| Propafenone | 1 2 (0-6.8) | 7 16 (5.2-27.3) | 16 37 (22.8-51.7) | 19 44 (29.3-59) | Seldom |
| Atenolol | 2 5 (0-10.9) | 7 16 (5.2-27.3) | 18 42 (27.1-56.6) | 16 37 (22.8-51.7) | Seldom |
| Bisoprolol | 5 12 (2-21.2) | 19 44 (29.3-59) | 11 26 (12.5-38.6) | 8 19 (7-30.2) | Sometimes |
| Carvedilol | 2 5 (0-10.9) | 9 21 (8.8-33.1) | 10 23 (10.6-35.9) | 22  51 (36.2-66.1) | Never |
| Celiprolol | 0 0 (0-0) | 0 0 (0-0) | 0 0 (0-0) | 43 100 (100-100) | Never |
| Labetalol | 0 0 (0-0) | 0 0 (0-0) | 2 5 (0-10.9) | 41 95 (89.1-100) | Never |
| Metoprolol | 6 14 (3.6-24.3) | 25 58 (43.4-72.9) | 5 12 (2-21.2) | 7 16 (5.2-27.3) | Sometimes |
| Nadolol | 4 9 (0.6-18) | 6 14 (3.6-24.3) | 6 14 (3.6-24.3) | 27 63 (48.3-77.2) | Never |
| Nebivolol | 0 0 (0-0) | 1 2 (0-6.8) | 4 9 (0.6-18) | 38 88 (78.8-98) | Never |
| Propranolol | 26 60 (45.9-75.1) | 12 28 (14.5-41.3) | 2 5 (0-10.9) | 3 7 (0-14.6) | Often |
| Sotalol | 4 9 (0.6-18) | 14 33 (18.6-46.6) | 6 14 (3.6-24.3) | 19 44 (29.3-59) | Seldom |
| Amiodaron | 12 28 (14.5-41.3) | 13 30 (16.5-44) | 12 28 (14.5-41.3) | 6 14 (3.6-24.3) | Sometimes |
| Dronedarone | 0 0 (0-0) | 0 0 (0-0) | 1 2 (0-6.8) | 42 98 (93.2-100) | Never |
| Diltiazem | 0 0 (0-0) | 0 0 (0-0) | 9 21 (8.8-33.1) | 34 79 (66.9-91.2) | Never |
| Verapamil | 1 2 (0-6.8) | 3 7 (0-14.6) | 10 23 (10.6-35.9) | 29 67 (53.4-81.4) | Never |
| Digoxin | 0 0 (0-0) | 5 12 (2-21.2) | 17 40 (24.9-54.1) | 21 49 (33.9-63.8) | Seldom |
| Ivabradine | 0 0 (0-0) | 2 5 (0-10.9) | 13 30 (16.5-44) | 28 65 (50.9-79.4) | Never |

**Supplementary Table 3**

Frequency of prescription of anticoagulants and antiplatelet drugs (n= 43)

| **Drug** | **Often n % (95% CI)** | **Sometimes n % (95% CI)** | **Seldom n % (95% CI)** | **Never n % (95% CI)** | **Median** |
| --- | --- | --- | --- | --- | --- |
| Acetylsalicylic Acid | 31 72 (58.7-85.5) | 10 23 (10.6-35.9) | 2 5 (0-10.9) | 0 0 (0-0) | Often |
| Clopidogrel | 2 5 (0-10.9) | 14 33 (18.6-46.6) | 9 21 (8.8-33.1) | 18 42 (27.1-56.6) | Seldom |
| Prasugrel | 0 0 (0-0) | 0 0 (0-0) | 2 5 (0-10.9) | 41 95 (89.1-100) | Never |
| Ticagrelor | 0 0 (0-0) | 0 0 (0-0) | 2 5 (0-10.9) | 41 95 (89.1-100) | Never |
| Apixaban | 3 7 (0-14.6) | 9 21 (8.8-33.1) | 11 26 (12.5-38.6) | 20 47 (31.6-61.4) | Seldom |
| Dabigatran | 0 0 (0-0) | 0 0 (0-0) | 2 5 (0-10.9) | 41 95 (89.1-100) | Never |
| Edoxaban | 0 0 (0-0) | 0 0 (0-0) | 1 2 (0-6.8) | 42 98 (93.2-100) | Never |
| Rivaroxaban | 3 7 (0-14.6) | 6 14 (3.6-24.3) | 7 16 (5.2-27.3) | 27 63 (48.3-77.2) | Never |
| Acenocoumarol | 7 16 (5.2-27.3) | 2 5 (0-10.9) | 4 9 (0.6-18) | 30 70 (56-83.5) | Never |
| Phenprocoumon | 12 28 (14.5-41.3) | 15 35 (20.6-49.1) | 11 26 (12.5-38.6) | 5 12 (2-21.2) | Sometimes |
